# Insulin-like growth factor-1 signaling in the tumor microenvironment: Carcinogenesis, cancer drug resistance, and therapeutic potential

**DOI:** 10.3389/fendo.2022.927390

**Published:** 2022-08-09

**Authors:** Armel H. Nwabo Kamdje, Paul F. Seke Etet, Maulilio J. Kipanyula, Lorella Vecchio, Richard Tagne Simo, Alfred K. Njamnshi, Kiven E. Lukong, Patrice N. Mimche

**Affiliations:** ^1^ Department of Physiological Sciences and Biochemistry, Faculty of Medicine and Biomedical Sciences, University of Garoua, Garoua, Cameroon; ^2^ Basic and Translational Research Unit, Center for Sustainable Health and Development, Garoua, Cameroon; ^3^ Neuroscience Lab, Faculty of Medicine and Biomedical Medicine, The University of Yaoundé l and Brain Research Africa Initiative (BRAIN), Yaoundé, Cameroon; ^4^ Department of Veterinary Anatomy and Pathology, College of Veterinary Medicine and Biomedical Sciences, Sokoine University of Agriculture, Morogoro, Tanzania; ^5^ Department of Biomedical Sciences, Faculty of Sciences, University of Ngaoundere, Ngaoundere, Cameroon; ^6^ Department of Biochemistry, Microbiology & Immunology, College of Medicine, University of Saskatchewan, Saskatoon, SK, Canada; ^7^ Division of Microbiology and Immunology, Department of Pathology, Molecular Medicine Program, University of Utah, Salt Lake City, UT, United States

**Keywords:** Insulin-like growth factor, tumor microenvironment, cancer, drug resistance, IGF-1, microenvironment

## Abstract

The tumor microenvironment fuels tumorigenesis and induces the development of resistance to anticancer drugs. A growing number of reports support that the tumor microenvironment mediates these deleterious effects partly by overexpressing insulin-like growth factor 1 (IGF-1). IGF-1 is known for its role to support cancer progression and metastasis through the promotion of neovascularization in transforming tissues, and the promotion of the proliferation, maintenance and migration of malignant cells. Anti-IGF therapies showed potent anticancer effects and the ability to suppress cancer resistance to various chemotherapy drugs in *in vivo* and *in vitro* preclinical studies. However, high toxicity and resistance to these agents are increasingly being reported in clinical trials. We review data supporting the notion that tumor microenvironment mediates tumorigenesis partly through IGF-1 signaling pathway. We also discuss the therapeutic potential of IGF-1 receptor targeting, with special emphasis on the ability of IGF-R silencing to overcome chemotherapy drug resistance, as well as the challenges for clinical use of anti-IGF-1R therapies.

## 1 Introduction

Tumorigenesis is a manifestation of malignant property in normal cells. According to emerging evidence from molecular studies, the tumor microenvironment uses key paracrine factors such as insulin-like growth factor 1 (IGF-1) to fuel tumorigenesis (1–3). Certainly, in the current global obesity epidemic, IGFs have attracted attention for their potential role as a link between cancer and insulin resistance syndrome (4–6), a metabolic syndrome of increasing incidence that encompasses hyper/hypoinsulinemia, hyper/hypoglycemia, dyslipidemia and hypertension, with a marked increase in risk to develop diabetes mellitus, cardiovascular diseases, and cancer (7–10). Notably, recent reports show that antidiabetic drugs, such as metformin, can mediate anticancer effects partly by silencing IGF-1 expression (9, 10).

Insulin-like growth factors (IGFs) are abundantly released by both malignant cells and the tumor microenvironment during tumorigenesis, metastasis, and the development of anticancer drug resistance (11–13). Silencing IGF-1R signaling has emerged as a promising strategy against cancer and for overcoming cancer drug resistance, as illustrated by a recent report of the antioxidant molecule vitamin D regulating apoptosis and autophagy and supporting the DNA repair process of cancer cells partly by silencing IGF-1R signaling and its downstream target, β-catenin (14). We review and discuss recent data supporting a key role for IGF-1 in tumor microenvironment-mediated tumorigenesis and cancer drug resistance development.

## 2 Tumor microenvironment and insulin-like peptides

### 2.1 Tumor microenvironment

The stroma is a collagenous connective tissue which, in healthy organs, includes: the extracellular matrix (ECM); mesenchymal stromal cells (MSCs) and derived cells such as connective tissue-producing fibroblasts; endothelial cells (blood vessels); as well as adipocytes, resident immune cells and other tissue-specific cells. The tumor microenvironment is an even more complex structure emerging during tumor progression due to the interactions of proliferating tumor cells with the host stroma. Typically, the tumor microenvironment is a stroma-like tissue with altered ECM components, newly formed blood vessels, infiltrating macrophages called tumor-associated macrophages (TAMs), and numerous cancer-affected fibroblasts called cancer-associated fibroblasts (CAF), which crosstalk with malignant cells to support tumorigenesis and are responsible for matrix remodeling (15, 16). Continuous interactions of the surrounding microenvironment with tumor cells result in the release or expression of paracrine factors, such as IGFs, which in turn initiate local signals fueling tumorigenesis and resistance to anti-cancer drugs (1–3).

### 2.2 Insulin-like peptides and receptors

Insulin-like peptides (ILPs), which include IGF-1, IGF-2, insulin, and seven relaxin-related peptides sharing the same basal fold in humans, are evolutionary conserved factors that play a central role in the regulation of energy metabolism, cell growth and proliferation, and neurotransmission (7, 8, 17).

**Figure 1:**
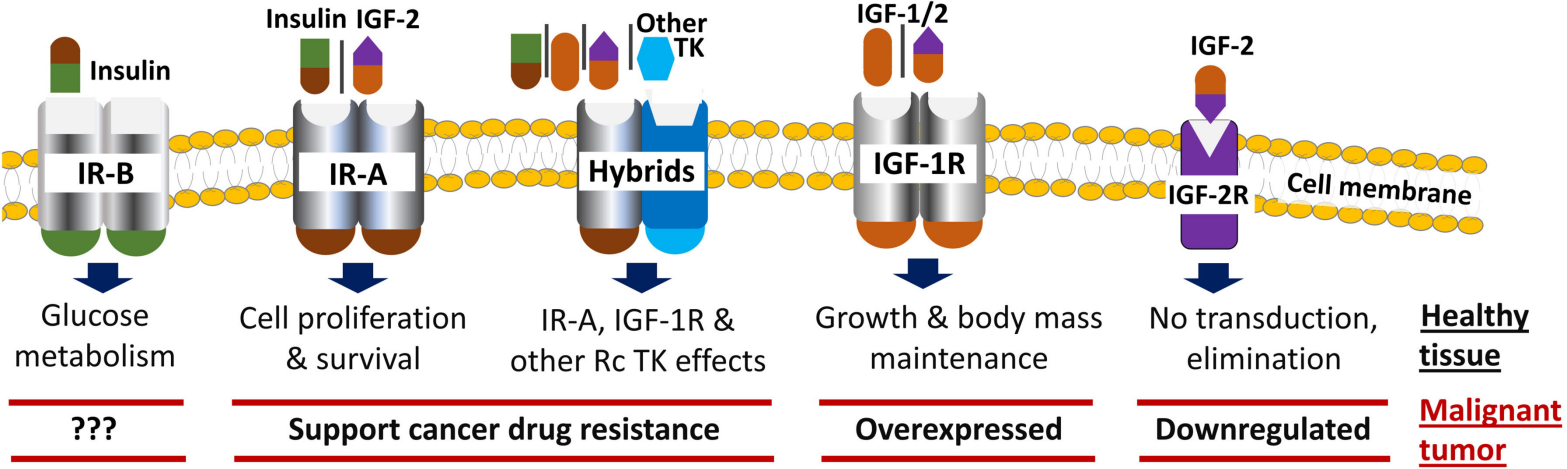
Insulin-like peptide receptors in healthy tissue and tumors.

ILP receptors include the IGF-2 receptor (IGF-2R) that clears IGF-2 from the cell surface without signal transduction and structurally related receptor tyrosine kinases, namely: insulin receptor isoform A (IR-A), which has a strong affinity for insulin and IGFs, and is involved in cell growth, proliferation and survival; IR-B which also has a strong affinity for insulin but not for IGFs, and which mediates differentiation and metabolic signals; IGF-1 receptor (IGF-1R), which plays a key role during growth and in maintaining body mass in adults; and hybrid receptors made of combinations of half IGF-1R and half insulin receptor isoforms or other receptor tyrosine kinases (18, 19) (**Figure 1**).

**Figure 2:**
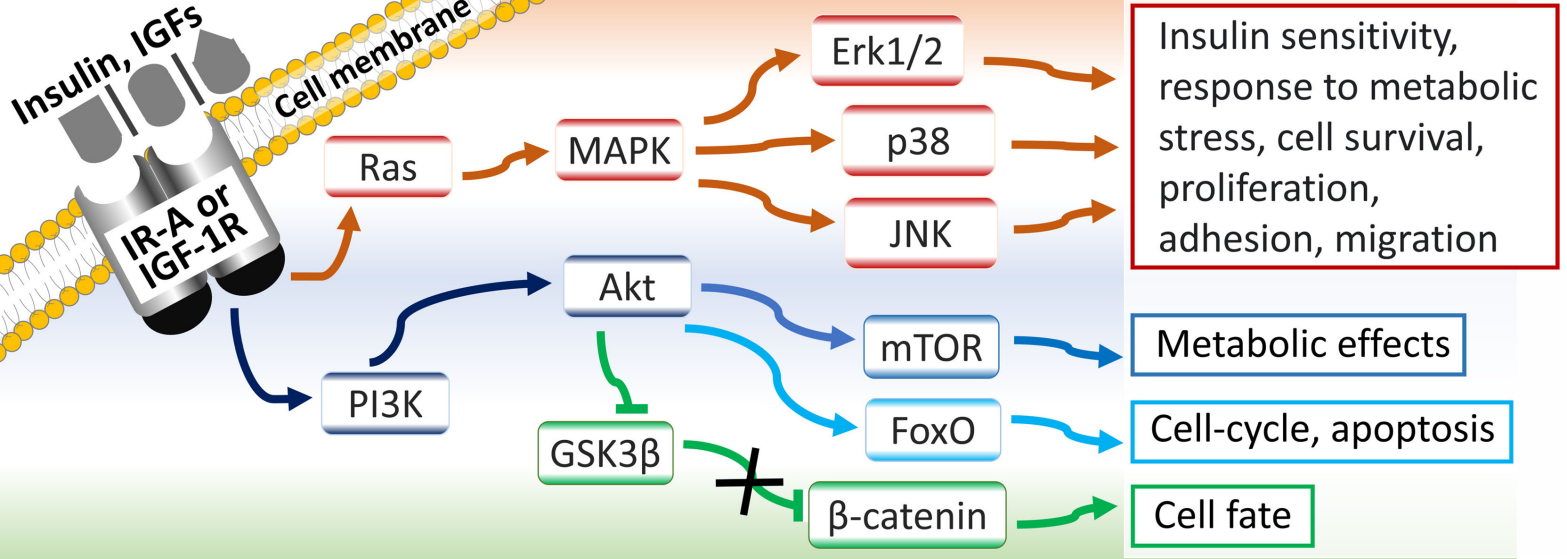
Signaling pathways and physiological roles of IR-A and IGF-1 receptors.

IR-A/B and IGF-1R mediate their effects through: Ras/mitogen activated protein kinase (MAPK)/extracellular signal-related kinase 1/2 (ERK-1/2) pathway; phosphoinositide 3-kinase (PI3K)/protein kinase B (Akt)/forkhead box O (FoxO) pathway; PI3K/Akt/mammalian target of rapamycin (mTOR) pathway; and PI3K/Akt-induced accumulation of the proto-oncogene β-catenin, *via* inactivation of its inhibitor glycogen synthase kinase 3β (GSK3β) (5, 14, 20) (**Figure 2**).

## 3 IGF-1R signaling: A key link between metabolic syndrome and tumorigenesis

There is controversy over whether the metabolic syndrome actually drives tumorigenesis or is just a passenger contributing on a case-by-case basis to this process. Epidemiological, clinical and experimental reports support that metabolic syndrome associated with insulin confers an increased risk of developing various cancers, including breast, endometrial, ovarian, colon and prostate cancers, as well as diseases associated with insulin resistance syndrome such as type 2 diabetes (21). Furthermore, available data suggest a key role for IGFs both as a link between insulin resistance syndrome and cancer, and as a driver of cancer drug resistance associated with metabolic syndrome. For instance, both diabetic and non-diabetic women with insulin resistance have higher risk to develop breast, endometrial and ovarian cancer, and results from studies in ovarian and cervical cancer suggest that the first-line type 2 diabetes drug and anti-insulin resistance agent metformin is a good candidate in combination therapies for these cancers (4, 9). Studies have shown that metformin has antitumor activity in the major gynecologic malignancies through a pleiotropic mechanism involving direct silencing of IGF-1R signaling and indirect silencing of this signaling pathway by targeting its downstream targets, including MAPK and mTOR (21, 22). Metformin also induced a G1-cell cycle arrest and apoptosis in EML_4_-ALK(+) lung cancer (H3122) cells partly through the modulation of IGF-1 expression (23).

On the same hand, to assess the mechanistic basis for the association between obesity and colorectal cancer, Bader and colleagues (2020) subcutaneously injected MC38 murine colon adenocarcinoma cells into high-fat diet-fed female, male, and ovariectomized female C57BL/6 mice. The findings revealed markedly accelerated subcutaneous tumor growth in obese females lacking ovarian hormones. The potential mechanisms driving obesity-mediated enhancement of cancer growth in this study mainly included TAMs-associated adipose inflammation and the release of adipose specific IGF-1 (6). In addition, obesity could be associated with cancer drug resistance. Obesity reduced the anti-cancer effects of the antidiabetic adiponectin receptor agonist AdipoRon in diet-induced obese mice with orthotopic pancreatic cancer through a mechanism involving IGF-1, IGF-1R, and ERK1/2 signaling (5), indicating the importance of weight loss in combating pancreatic cancer in obese patients.

However, despite such observations and the well-established epidemiological link between obesity and the severity of prostate cancer (11, 12), some reports raise questions about t the actual role of obesity in the progression of prostate cancer. For example, Lo and colleagues (2016) reported that obesity failed to promote tumorigenesis in localized patient-derived prostate cancer xenografts in severe combined immunodeficiency (SCID) mice (13). In this study, obesity did not promote prostate tumorigenicity and no differences between lean and obese mice were observed in tumor progression and in the expressions of homeobox protein Nkx-3.1, androgen receptor, and prostate-specific antigen. Taken together, these observations confirm that obesity drives tumorigenesis in at least some cases of prostate cancer. Mechanistic studies investigating biological factors linking obesity and carcinogenesis, and in particular the role of IGF-1R signaling in the development of tumors in the context of obesity, may reveal populations at risk for obesity-driven tumorigenesis, as well as potential therapeutic targets for cancer therapy.

## 4 Tumor microenvironment, tumorigenesis and drug resistance

Tumor microenvironment highly active cells, and in particular adipose tissue and TAMs, are potential determinant for a driving role of obesity and other risk factors in cancer development.

### 4.1 Tumor-associated macrophages

Various reports support that TAMs promote tumorigenesis through IGF-dependent mechanisms (20, 24–26). For instance, in a study, assessing TAM ability to affect the malignant phenotype of human hepatoma Huh-7 cells, TAMs promoted the migration, invasion, and epithelial–mesenchymal transition (EMT) of Huh-7 cells through a mechanism requiring activation of the Gli2/IGF-2/ERK1/2 signaling pathway and the resulting secretion of transforming growth factor beta 1 (TGF-β1) (24). Moreover, IGF−1 and IGF−2 secreted by M2−like TAMs increased markedly the invasive ability and stemness of anaplastic thyroid carcinoma cells *in vitro*, as revealed by transwell and sphere formation assays, through the activation of the PI3K/AKT/mTOR pathway (20). The importance of the latter signaling pathway was also illustrated by other recent reports, including: a study where the novel small molecule acetonitrile derivative monepantel effectively suppressed ovarian cancer cells’ growth, proliferation, and colony formation by down-regulating IGF-1R and inhibiting its mTOR/p70S6K downstream target in these cells (25); and another study which showed that chemotherapy and radiotherapy resistance is mediated in ovarian cancer by hypoxia-inducible factor 1 alpha (HIF-1α) and IGF-1-mediated upregulation of reactive oxygen species (ROS) inducer NADPH oxidase 4 (NOX4) (26). Furthermore, the translocation to the plasma membrane of 78-kDa glucose-regulated protein GRP78, which facilitates M2 macrophage polarization of TAMs and tumor growth in lung cancer, was induced by IGF-1R signaling, and concomitant IGF-1 blockade and GRP78 knockdown in TAMs suppressed M2 macrophage-induced maintenance, proliferation, and migration of lung cancer cells (27).

### 4.2 Adipose tissue

Early studies strongly support that periprostatic adipose tissue, particularly under metabolic syndrome-associated inflammation, is a key player in the development of prostate cancer, through the IGF-1 axis, adipokines, and sex hormones (28, 29). Overall, there is a crosstalk between cancer cells and the periprostatic adipose tissue, locally-produced adipokines support the development of the tumor microenvironment, and fatty acid-binding protein 4 (FABP4) released by adipocytes would constitute an energy source for tumor cell invasion (28, 30). In a recent *in vitro* study using human prostate cancer cell lines DU145 and PC3, periprostatic adipose tissue promoted prostate cancer resistance to the chemotherapy drug docetaxel through a mechanism involving paracrine IGF-1 upregulation of the β-tubulin isoform of tubulin Beta 2B Class IIb (TUBB2B) gene (31). In this study, the IGF-1 receptor inhibitor AG1024 increased the response of resistant cancer cells to docetaxel and decreased the expression of TUBB2B, highlighting the potential of targeting the IGF-1 axis as a novel therapeutic strategy. aimed at stopping the support of periprostatic adipocytes to invading prostate cancer cells.

### 4.3 Fibroblasts and stellate cells

**Figure 3:**
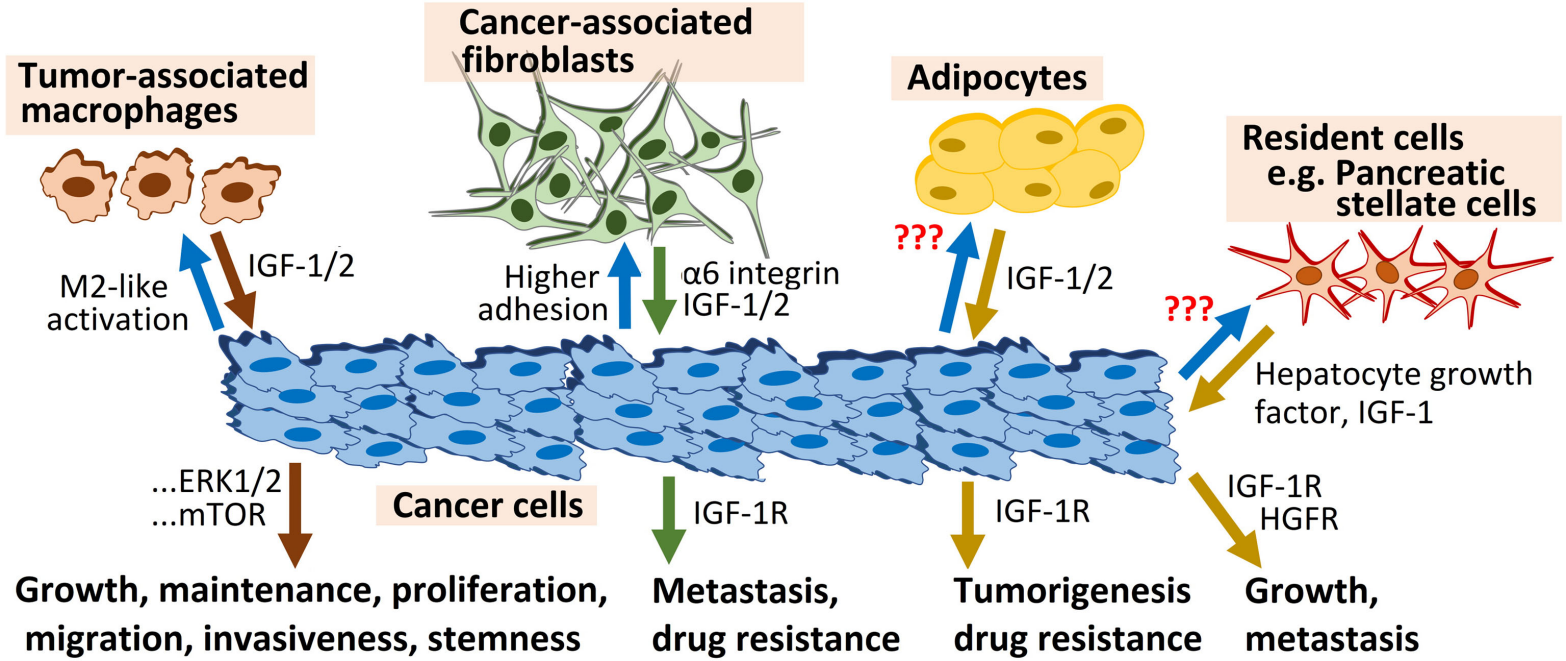
IGF-1R signaling triggered by the microenvironment fuels tumorigenesis.

Fibroblasts and pancreatic stellate cells also use IGF-1R signaling to support tumorigenesis and cancer drug resistance. A recent report showed that higher adhesion to primary lung fibroblasts is associated with escalation of intrinsic and acquired chemoresistance in epithelial ovarian cancer cells, and is largely governed by α6 integrin-IGF-1R dual signaling axes (32). Interestingly, in this study aimed at assessing the mechanistic role of IGF-1R signaling in the regulation of organ-specific metastasis of platinum-taxol-resistant A2780 EOC cells in orthotopic xenograft mouse models, IGF-1R silencing abrogated organ-specific metastasis of EOC cells augmented by acquired chemoresistance, and enhanced lung homing of the late-stage chemoresistant cells. On the same hand, the metastasis suppressor N-myc downstream regulated gene 1 (NDRG1) interrupted tumorigenic bidirectional crosstalk between pancreatic cancer cells and stromal pancreatic stellate cells, major contributors to local tumor growth and metastasis, partly through inhibition of IGF-1 and other potent mediators of cell migration, including hepatocyte growth factor (HGF) (33) (**Figure 3**).

### 4.4 Extracellular matrix and blood vessels

*Plasmodium* infection ability to boost immunity, inhibit tumor progression, and prolong survival in murine Lewis lung carcinoma, triple negative breast cancer, and hepatocellular carcinoma models was reported (34–36). Interestingly, mechanistic studies revealed that *Plasmodium*-derived hemozoin that accumulated in TAMs abrogated IGF-1R signaling partly by silencing its downstream targets PI3K and MAPK, causing decreased expression of tumor angiogenesis facilitator matrix metalloprotease 9 (MMP-9) in TAMs, and in turn leading to decreased tumor angiogenesis and TAM infiltration (34).

In addition, the small leucine-rich repeat proteoglycan of bone extracellular matrix tissue biglycan, which is correlated with an aggressive phenotype of osteosarcoma, supported tumor growth and induced resistance to chemotherapy drug doxorubicin by forming a complex with IGF-1R leading to activation of the IGF-1R signaling pathway in human osteosarcoma cell line MG63 (37). Similarly, miR-520b promoted doxorubicin sensitivity in breast cancer cells through downregulation of IGF-1R, i.e., abrogating tumor development, metastasis, and resistance to chemotherapy induced by IGF-1R (38). Moreover, IGF-1 released by the tumor microenvironment supported the development of drug resistance by sustaining the prompt regrowth of resistant tumor and by driving the remodeling of the tumor vasculature through activation of IGF-1R signaling on endothelial cells in a xenograft mouse model of melanoma (39). Of particular interest for the development of therapeutic strategies for preventing tumor relapse, blockade of IGF-1R with small molecules disrupted vascular reconstruction and delayed tumor relapse (39).

## 5 Therapeutic potential of IGF-1-R targeting in cancer

### 5.1 IGF-1R silencing can abrogate cancer drug resistance *in vitro*

**Figure 4:**
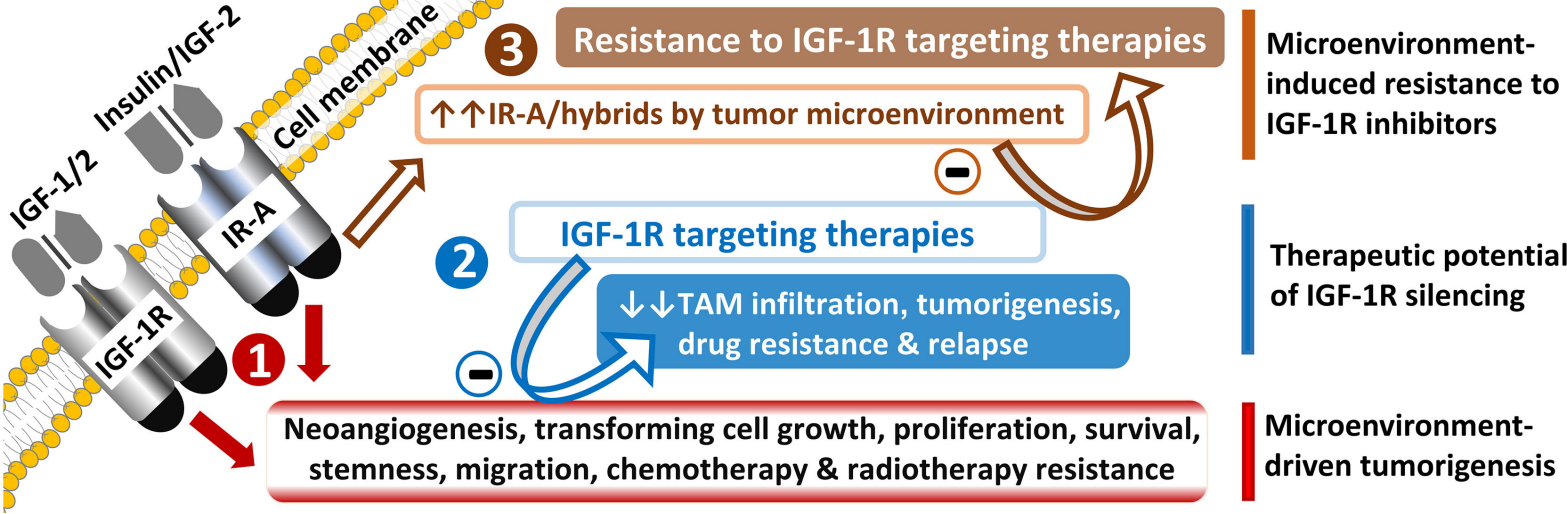
Potential and a challenge of IR-A/IGF-1R targeting in tumors.

It appears from the data presented above that IGF-1-R signaling targeting could be an excellent therapeutic strategy in cancer, particularly in chemotherapy resistant cases (**Figure 4**). Other examples include the recently reported ability of nuclear IGF-1R to drive chemotherapy resistance in various types of cancer, and notably, through interactions with nuclear mitotic apparatus (NuMA) protein and regulating p53−binding protein 1 (53BP1) −dependent DNA double−strand break repair in colorectal cancer (40). Resistance to osimertinib, a third-generation tyrosine kinase inhibitor targeting EGF receptor used as first-line therapy for patients with non-small cell lung cancer (NSCLC) harboring mutant EGFR, is mediated by IGF-1R signaling activated by IGF-2 in NSCLC cell lines (41). The IGF-1R axis also contributed to NSCLC resistance to folate analog pemetrexed *in vitro* (42). Moreover, the invasion of cancer cells and resistance to multi-kinase inhibitors regorafenib and sorafenib were also associated with increased IGF-1R signaling in metastatic colorectal cancer and hepatocellular carcinoma (43–45).

Besides, Dawson and colleagues (2021) reported that androgen deprivation therapy given to prostate cancer survivors to treat recurrent cancer is associated with cancer cachexia despite resistance training, which counteracts both muscle and physical function loss in other settings. This loss partly occurred due to lack of reduction in resting mRNA expression of myostatin and elevations in mRNA expression of IGF-1, PGC-1α4 and myogenin, which are generally expected after resistance training (46). Thus, new therapeutic strategies should include targeting the IGF axis in malignant cells and tumor microenvironmental drivers in prostate cancer.

### 5.2 IGF-1-R inhibitors vs. drug resistant cancers

Clinical and experimental evidence suggests strong potential for IGF-1-R antagonists to overcome resistance to anti-cancer chemotherapy drugs (40, 47–49). For instance, manuka honey restored the ability of metastatic colorectal cancer first-line drug 5-fluorouracil to abrogate resistance to apoptosis of cancer stem-like cells derived from the HCT-116 colon adenocarcinoma cell line by a pleiotropic mechanism involving the downregulation of IGF-1, IGF-2, IGF-1R, as well as other inhibitors of apoptosis, such as: inhibitors of apoptosis protein (IAP) family members survivin, livin, and XIAP; and heat shock proteins HSP-27, HSP-60, and HSP-70 (47). Notably, another recent report suggested that chemoresistance of colorectal cancer cells to the DNA replication inhibitor 5-fluorouracil results from a potent cell survival mechanism based on sustained translation of IGF-1R mRNA and impaired ribosomal function (48).

Also sustaining this hypothesis, other authors observed that modulation of IGF-1R-associated genes promoted regulatory clustering as well as stemness and chemoresistance in cells from colorectal cancer patients and mouse models, and was facilitated by a master transcriptional regulator responsive to ribosomal dysfunction, PR domain zinc finger protein 1 (PRDM1) (49). Suppression of SNAIL2, a zinc finger protein member of the SNAIL superfamily of epithelial-mesenchymal transition-inducing transcription factors (EMT-TFs) commonly overexpressed in pancreatic cancer patients, restored sensitivity of pancreatic cancer cell lines KLM1 and KMP5 to chemotherapy drug gemcitabine through an IGF binding protein 2 (IGFBP2)-dependent mechanism *in vitro* and *in vivo* (50).

Interestingly, in a recent study using resistant colon cancer stem-like cells and mouse models, combination therapy of regorafenib with two antagonists of IGF-1-R, namely aspirin and the selective dual insulin receptor and IGF-1R kinase inhibitor linsitinib (OSI-906), abrogated tumor resistance to regorafenib and restored the drug’s ability to induce apoptosis in colon cancer stem-like cells and disease activity index (51). Linsitinib also restored the sensitivity of ovarian clear cell carcinoma (OCCC) cells to the chemotherapy drug cisplatin by silencing the IGF-1R/AKT signaling pathway (52). Notably, in this study, OCCC which has greater disease aggressiveness and resistance to chemotherapy than epithelial ovarian cancer, the most common type of ovarian cancer, also showed higher levels of IGF-1 (52). These observations indicate that the strength of responses of chemotherapy-resistant cancers to anti-IGF agents will depend on the extent of IGF-1R signaling in tumors.

## 6 Challenges for clinical use of IGF-1R-silencing agents

### 6.1 Toxicity of IGF-1R antagonists

Clinical trials of a large number IGF-1/2, IGF-1R, IRS-1, PI3K and mTOR antagonists have been abandoned due to limited anti-cancer activity, or to the toxicity of these agents (41, 53), further supporting the need to continue studies on LL6 and other molecules that are well tolerated in mice and effective at doses that are not toxic to normal cells (54). Notably, serious adverse effects (SAE) in clinical trials may result from dysfunctions of tissues that are highly dependent on IGF-1R signaling for their physiological functions, including muscle, adipose, and bone tissues (46, 55). The most common adverse events (AE) reported included skin-related toxicities, dehydration, diarrhea, asthenia, rash, hyperglycemia, hypertension, neutropenia, thrombocytopenia, anemia, hepatic dysfunction, proteinuria (56–58), and hearing loss (59, 60), with non-fatal SAE occurred in 5-25% of patients, as well as a few treatment-related deaths (61).

Various study on IGF-1R, IGF-1 and IGF-2 expressions reported findings usable for useful patient stratification in future trials with inhibitors of the IGF-1R signaling pathway, to sort out those that may benefit the most from anti-IGF-1R therapies, limiting exposure of other patients to these agents (62, 63).

### 6.2 Need for IGF-1R targeting agents with higher specificity

The drug candidates targeting IGF-1R signaling that are under clinical trial investigation include: IGF ligand inhibitors, which are monoclonal antibodies targeting IGF-1/2; IGF-1R antagonists, which encompass some antibodies and kinase inhibitors; as well as insulin receptor substrate 1 (IRS-1) inhibitors, PI3K inhibitors, and mTOR inhibitors alone or in combination (25, 52, 64, 65). These agents are of the same classes of drugs abandoned due to their high toxicity (41, 53).

Interestingly, various reports, including a study aimed at developing new insulin analogues thermally more stable and with more physiological profiles than human insulin provided evidence for the possibility to develop IR-A/IGF-1R targeting agents with higher specificity (66). In this study, chemical engineering of insulin analogues allowed the development of an insulin analogue with more than 3-fold-enhanced binding specificity for the metabolic IR-B isoform (67), allowing to prevent the pro-tumorigenic effects of insulin mediated by IR-A and hybrids with IGF-1R or other receptor tyrosine kinases (68, 69). We propose that comparable approaches aimed at developing agents targeting IGF-1R downstream signaling molecules specifically implicated in each type of cancer on a case-by-case basis could have better anticancer resistance effects with less toxicity. Interestingly, on the same hand, emerging reports suggest that the expressions of insulin and IGF receptor isoforms vary according to tissue types and ligand bioavailability (70) and that there are therapeutic selective dependencies for distinct subtypes of PI3K pathway altered in cancer (71).

### 6.3 Cancer resistance to anti-IGF-1R therapies

A body of literature has reported evidence of resistance to anti-IGF-1R therapies. Non-competitive overactivation of IGF-1R signaling (through mutations) support NSCLC resistance to xentuzumab (BI 836845), a monoclonal antibody targeting both IGF-1 and IGF-2 (72). In addition, drug resistance is also conferred by Src and AXL membrane tyrosine kinases (73). To develop kinase inhibitors that simultaneously target Src, AXL and IGF-1R activities, Lee and colleagues (2021) synthesized a series of compounds based on phenylpyrazolo[3,4-d]pyrimidine conjugation with 2,4-bis-arylamino-1,3-pyrimidines (I2). LL6, the most promising novel small molecule kinase inhibitor obtained, induced apoptosis and suppressed the colony-forming capacities of various drug-resistant NSCLC cell lines, suppressed NSCLC cell migration, suppressed NSCLC xenograft tumor growth and Lewis lung carcinoma allograft tumor metastasis in mice, and markedly reduced the number and burden of lung tumors in KrasG12D/+ transgenic mice, with low toxicity (54). Further studies of LL6 and other kinase inhibitors simultaneously targeting Src, AXL and IGF-1R activities could provide a new avenue for the treatment of drug-resistant NSCLC.

Similarly, other small molecule inhibitors and antibody-drug conjugates are being tested in other types of cancers where the IGF-1R/PI3K and AXL signaling pathways have also recently attracted attention, including in primary bone cancers where they play a major role in oncogenesis, cell differentiation and fate, metastasis, and drug resistance (53). Targeting of IGFs, IGF-1R and its downstream targets by antibodies has also been proposed as a strategy to silence IGF-1R signaling with less resistance. An example is B003-2A, a recently developed IGF-1R anti-idiotypic antibody antagonist, which abrogated the IGF-1R-mediated proliferation of ovarian cancer cells *in vivo* and *in vitro* and overcame resistance to cisplatin in ovarian cancer cell lines (64, 74).

### 6.4 IGF-1R tumorigenic interactions with other receptor tyrosine kinases

#### 6.4.1 IGF-1R and FGFR1 axes

Manipulating the IGF-1R signaling pathway has been viewed by many scholars as a potential breakthrough in anticancer therapies. Disappointing *in vivo* effects of anticancer molecules mediating strong effects *in vitro* through IGF-1R silencing were observed, possibly due to cancer cell resistance mediated by compensatory mechanisms activating IGF-1R downstream targets. A study addressing why the IGF-1R pathway silencing through IGF-1R fusion protein IGF-Trap-induced IGF-1/2 bioavailability reduction, which was a promising anticancer strategy *in vitro* failed in nude mice xenotransplanted with the human triple negative breast cancer MDA-MB-231 cells, found that these cells developed resistance to IGF-targeted therapy by increasing the activation of another receptor tyrosine kinase, fibroblast growth factor receptor 1 (FGFR1) (75). FGFR1 silencing with the FGFR1-specific tyrosine kinase inhibitor PD166866 increased the sensitivity of MDA-MB-231 cells to IGF-Trap treatment *in vivo*.

Breast cancer cells can develop metformin resistance by triggering IGF-1R target insulin receptor substrate-1 (IRS-1)/ERK signaling through overactivation of FGFR1; IRS-1 acts as a critical crosstalk mediator between IGF-1R and FGFR1 pathways, encompassing a feedback loop between IRS-1 and MAPK/ERK (3). Thus, metformin sensitivity could be restored by targeting the FGFR1. Moreover, a mechanistic study addressing the development of resistance to the anti- human epidermal growth factor receptor 2 (HER2) monoclonal antibody trastuzumab in HER2-positive (HER(+)) gastric cancer found that endoplasmic reticulum stress induces resistance to trastuzumab in HER2(+) gastric cancer cells by activating IGF-1R and FGFR1 signaling pathways, through a microRNA (miR)-301a-3p-dependent mechanism (76). Trastuzumab resistance was also partly mediated by the IGF-1R pathway in HER2(+) breast cancer cells (1, 2).

#### 6.4.2 Deleterious interactions with other growth factors

Further supporting the complexity of crosstalk between IGF-1R and other tyrosine kinase receptor pathways, unclear synergistic actions of EGF, PDGF, TGF-β, and IGF induce stemness, cancer progression and metastasis, drug resistance, and tumor relapse in various cancer types (33, 39, 53, 77). A study using a panel of cell lines of high-risk neuroblastoma, a cancer characterized by increased MAPK signaling, found drastic variations in sensitivity to serine/tyrosine/threonine kinase inhibitors between these cell lines (7). Surprisingly, mathematical modeling of these variations revealed that MAPK signaling negative feedback *via* IGF-1R reactivated MAPK signaling to mediate cancer cell drug resistance, highlighting the concomitant targeting of MEK and IGF-1R/MAPK signaling pathways as a potential therapeutic strategy in high-risk neuroblastoma. Furthermore, a crosstalk between insulin-induced ERK and epidermal growth factor (EGF) favored tumorigenesis by triggering the expression of anti-tumor immunity inhibitor programmed death-ligand 1 (PD-L1) in a cancer common in individuals with insulin resistance, pancreatic ductal adenocarcinoma (78).

Plausible explanations for incongruence of findings on the role of IGF-1R include the complex compensatory mechanisms mediated by other tyrosine kinase receptor pathways and interspecies differences. For instance, the addition of ganitumab, a monoclonal antibody targeting IGF-1R, to carboplatin/paclitaxel chemotherapy in patients with primary epithelial ovarian cancer did not improve progression-free survival in a phase II multicenter controlled trial (65), despite the promising results of IGF-1R inhibition in various preclinical studies (25, 32, 64). In addition, although both receptors facilitate chemoresistance to the selective estrogen receptor modulator (SERM) tamoxifen in breast cancer, resistance to tamoxifen is characterized by decreased expression of IGF-1R and increased EGFR expression, and more surprisingly, no detectable difference was found in EGFR expression between breast cancer cells resistant and sensitive to tamoxifen when measured with cytometry (79). Future studies should characterize the interactions between tyrosine kinase receptors in cancer, considering the implications for overcoming cancer resistance to chemotherapy drugs.

## 7 Concluding remarks

IGFs are abundantly released by the tumor microenvironment and cancer cells to support tumor neovascularization and the maintenance, proliferation, and migration of cancer cells. During the invasion of tumor-promoting cells in various cancer types: microenvironment adipose tissue releases factors like IGF-1 and adipokines to support the development of the tumor microenvironment, and to promote cancer drug resistance; TAMs promote EMT and malignant cell stemness, migration, and invasion through a mechanism requiring IGF-1R/ERK1/2 signaling and the secretion of TGF-β1; and higher adhesion to fibroblasts, which is associated with escalation of intrinsic and acquired chemoresistance in lung cancer, for instance, is largely governed by α6 integrin-IGF-1R dual signaling axes. Taken together, these observations support that IGF-1 is a key player in tumor microenvironment-mediated tumorigenesis, metastasis, and anti-cancer drug resistance. Resistance to anti-IGF therapies is mediated by other receptor tyrosine kinases and their downstream targets through complex interactions. Future mechanistic studies investigating these interactions will improve our understanding of receptor tyrosine kinase functioning and provide therapeutic targets to overcome cancer resistance to various chemotherapy drugs.

## Author contributions

All authors listed have made a substantial, direct, and intellectual contribution to the work and approved it for publication.

## Acknowledgments

The authors thank colleagues from their institutions for proofreading the manuscript.

## Conflict of interest

The authors declare that the research was conducted in the absence of any commercial or financial relationships that could be construed as a potential conflict of interest.

## Publisher’s note

All claims expressed in this article are solely those of the authors and do not necessarily represent those of their affiliated organizations, or those of the publisher, the editors and the reviewers. Any product that may be evaluated in this article, or claim that may be made by its manufacturer, is not guaranteed or endorsed by the publisher.
